# Roles of ATM and ATR-Mediated DNA Damage Responses during Lytic BK Polyomavirus Infection

**DOI:** 10.1371/journal.ppat.1002898

**Published:** 2012-08-30

**Authors:** Mengxi Jiang, Linbo Zhao, Monica Gamez, Michael J. Imperiale

**Affiliations:** 1 Department of Microbiology and Immunology and Comprehensive Cancer Center, University of Michigan Medical School, Ann Arbor, Michigan, United States of America; 2 Graduate Program in Cancer Biology, University of Michigan Medical School, Ann Arbor, Michigan, United States of America; University of Pittsburgh, United States of America

## Abstract

BK polyomavirus (BKPyV) is an emerging pathogen whose reactivation causes severe disease in transplant patients. Unfortunately, there is no specific anti-BKPyV treatment available, and host cell components that affect the infection outcome are not well characterized. In this report, we examined the relationship between BKPyV productive infection and the activation of the cellular DNA damage response (DDR) in natural host cells. Our results showed that both the ataxia-telangiectasia mutated (ATM)- and ATM and Rad-3-related (ATR)-mediated DDR were activated during BKPyV infection, accompanied by the accumulation of polyploid cells. We assessed the involvement of ATM and ATR during infection using small interfering RNA (siRNA) knockdowns. ATM knockdown did not significantly affect viral gene expression, but reduced BKPyV DNA replication and infectious progeny production. ATR knockdown had a slightly more dramatic effect on viral T antigen (TAg) and its modified forms, DNA replication, and progeny production. ATM and ATR double knockdown had an additive effect on DNA replication and resulted in a severe reduction in viral titer. While ATM mainly led to the activation of pChk2 and ATR was primarily responsible for the activation of pChk1, knockdown of all three major phosphatidylinositol 3-kinase-like kinases (ATM, ATR, and DNA-PKcs) did not abolish the activation of γH2AX during BKPyV infection. Finally, in the absence of ATM or ATR, BKPyV infection caused severe DNA damage and aberrant TAg staining patterns. These results indicate that induction of the DDR by BKPyV is critical for productive infection, and that one of the functions of the DDR is to minimize the DNA damage which is generated during BKPyV infection.

## Author Summary

BK polyomavirus (BKPyV) is a human pathogen that establishes a persistent sub-clinical infection in healthy humans. When patients are immunosuppressed, particularly in kidney and bone marrow transplantation, the virus can reactivate and result in severe disease. BKPyV-related disease has risen due to the use of newer immunosuppressive regimens and an increase in the number of transplants performed each year. We are interested in understanding the interactions between BKPyV and host cell components or pathways, with the aim of developing more BKPyV-specific antiviral treatment options. In this study we characterized the relationship between BKPyV infection and the cellular DNA damage response (DDR), a signaling cascade that is initiated by cells to repair damaged DNA molecules. Our study indicated that BKPyV activates and hijacks the DDR to facilitate its infection and that various components of the DDR may play distinct roles during this process. These data suggest that the DDR may provide a potential host target to control BKPyV reactivation in transplant recipients.

## Introduction

BK polyomavirus (BKPyV) was first isolated in 1971 from a renal transplant patient [1] and has gained much interest in the past two decades due to its disease prevalence in immunocompromised patients [2]. Infection with BKPyV is ubiquitous in healthy individuals but does not lead to any known clinical disease. Under immunosuppressed conditions, especially in renal transplant and bone marrow transplant recipients, the virus can reactivate from a persistent state to lytic infection, which results in severe disease including polyomavirus-associated nephropathy (PVAN) and hemorrhagic cystitis (HC), respectively [2]. Unfortunately, there is currently no FDA-approved, specific anti-BKPyV drug available for treating these diseases. The common approach to control BKPyV reactivation is palliative care for HC patients, or combining immunosuppression reduction with drugs that inhibit viral DNA replication for PVAN, although there are often conflicting outcomes with these treatment options [3].

Much of the knowledge about the polyomavirus lytic life cycle comes from research performed on Simian Virus 40 (SV40). The viral genome, a circular double-stranded DNA molecule, is delivered into the nucleus. Following nuclear entry, early proteins including the large T antigen (TAg) are expressed. TAg sets up the host environment for viral DNA replication by inducing cells into S phase and, at the same time, inhibiting the p53-dependent apoptotic pathway [4]. Initiation of viral DNA replication requires the concerted efforts of TAg, replication protein A (RPA), DNA polymerase alpha-primase (Pol-prim), and topoisomerase I [5]. Newly replicated viral DNA is encapsidated by the capsid proteins VP1, VP2, and VP3, and this is followed by viral egress and cell lysis, thus completing the life cycle. Although SV40 is well-studied, there are differences between it and BKPyV. There is still much that is unknown with regard to the interaction between host nuclear components and viral factors during BKPyV replication. Identification and characterization of these interactions is extremely important, since it may reveal novel anti-viral therapeutic targets.

The DNA damage response (DDR) is emerging as a cellular process that is targeted by a number of DNA and RNA viruses [6], [7]. The DDR involves signaling cascades that are initiated when cells experience various types of DNA damage, and it coordinates many cellular processes including cell cycle arrest, chromatin modification, and DNA repair to allow cells to repair the damaged DNA [8], [9]. This response is largely orchestrated by two major phosphatidylinositol 3-kinase-like kinases (PI3KKs): ataxia telangiectasia mutated (ATM), and ATM and Rad3-related (ATR) kinase. ATM mainly responds to double-stranded breaks (DSBs) resulting from conditions such as ionizing irradiation (IR). The Mre11-Rad50-NBSI (MRN) protein complex serves as a sensor for DSBs, and is crucial for the activation of ATM upon DSB damage [10], [11]. ATR, on the other hand, is activated by single-stranded DNA lesions and is important for resolving replication stress from conditions such as stalled replication forks and ultraviolet (UV) light [8]. Both kinases, when activated, can phosphorylate numerous downstream targets that are involved in DNA repair and cell cycle arrest, including Chk1, Chk2, and a histone variant H2AX [8], [12]. The phosphorylated H2AX (serine 139, referred to as γH2AX) is considered a hallmark of the DDR, and it is crucial for recruiting and maintaining downstream mediator and repair proteins to sites of damage [13], [14].

There is accumulating evidence suggesting that the DDR is closely linked to polyomavirus infections. Mouse polyomavirus (MPyV) infection increases phosphorylated ATM (pATM), and using either an ATM inhibitor or ATM-deficient cells, it has been demonstrated that an ATM-mediated DDR is required for MPyV replication [15]. ATM phosphorylates SV40 TAg, and knockdown of ATM decreases the level of TAg-pS120 and viral DNA synthesis [16]. The ATM-mediated DDR has also been reported to be required for SV40 infection, and SV40 infection is found to lead to the proteasome-dependent degradation of MRN complex [17], [18]. Another human polyomavirus, JC polyomavirus (JCPyV), also activates the ATM DDR. It is suggested that DDR activation induces G2 arrest to promote JCPyV replication [19]. The contribution of the ATR-mediated DDR to polyomavirus infection is less clear. Although knockdown of ATR during JCPyV infection results in a decrease in TAg levels, ATR knockdown does not seem to affect SV40 DNA replication [18]. This lack of effect on viral replication has been attributed to incomplete knockdown of ATR and kinase redundancy [18]. Using a cell line that expresses a dominant negative form of ATR, another group showed that ATR is required for the activation of ATR-Δp53 signaling pathway during SV40 infection [20]. The activation of this pathway is believed to lead to intra-S checkpoint activation and promote TAg- Pol-prim complex formation [20]. Finally, a recent microarray analysis has shown that several genes that are involved in DNA damage repair are also upregulated with BKPyV infection, indicating that the DDR may be important during BKPyV infection [21].

In this study we examined the roles of both the ATM and ATR-mediated DDR during lytic BKPyV infection in a primary human renal proximal tubule epithelial (RPTE) cell culture model [22]. Our results showed that BKPyV activates both branches of the DDR. Using small interfering RNA (siRNA) knockdowns, we demonstrated that ATM and ATR each contribute to DDR activation caused by BKPyV infection. Our data also clearly suggested the importance of ATR during lytic BKPyV infection, and more importantly, that both proteins function additively to ensure efficient viral DNA replication and synergistically to affect production of viral progeny. In the absence of either kinase, severe DNA damage accumulated during BKPyV infection, indicating that part of the roles that ATM and ATR play is to repair DNA damage caused by BKPyV infection.

## Results

### BKPyV infection activates an ATM-mediated DDR

**Figure 1:**
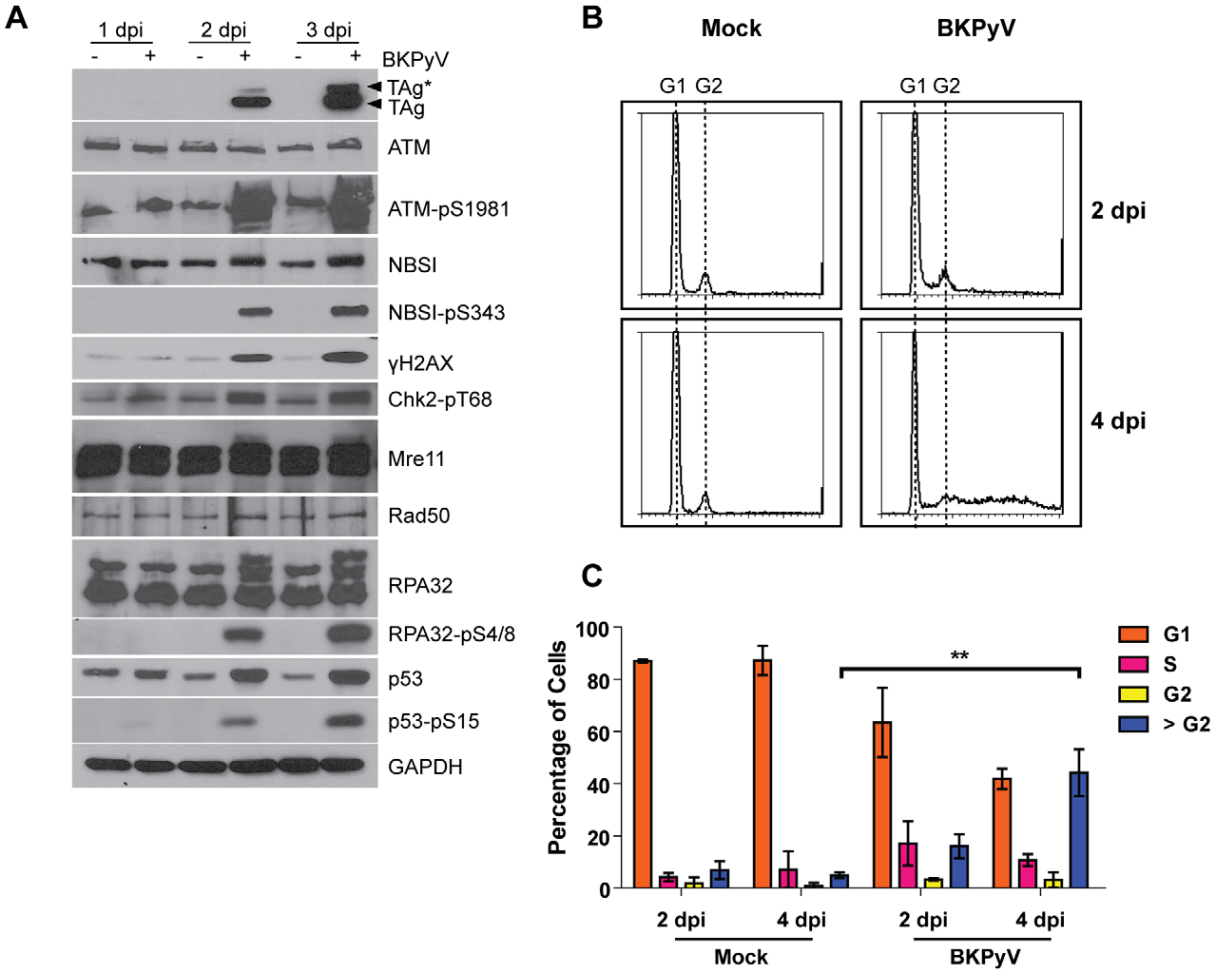
BKPyV infection activates the ATM-mediated DDR and induces polyploidy. (A) RPTE cells were mock infected or infected with BKPyV at an MOI of 5 IU/cell. Total proteins were harvested at the indicated times post infection and probed for the indicated proteins by Western blotting. The two filled arrowheads point to TAg and TAg* as described in Results. (B) RPTE cells were mock infected or infected with BKPyV at an MOI of 5 IU/cell. Cells were fixed at the indicated times post infection and stained with propidium iodide for flow cytometry analysis. Shown are representative histograms of PI intensity. The positions of G1 and G2 peaks are marked. (C) The percentage of cells in each cell cycle phase was calculated using ModFit LT software. Each bar represents the average from three independent experiments and the error bars are the standard deviation (SD) values. **, p<0.01 (all statistical analyses were performed using a two-tailed and unpaired Student's t-test).

To investigate the relationship between BKPyV and the DDR, we began our studies with the ATM-mediated branch of DDR, because it has been implicated in many polyomavirus infections [15]–[18]. To examine whether BKPyV infection activates an ATM-mediated DDR, we harvested total protein lysates from mock- and BKPyV-infected RPTE cells over a 3-day time course. The proteins were immunoblotted for markers that are indicative of ATM-mediated DDR activation (Figure 1A). The gradual increase in the viral early protein TAg allowed us to monitor the progression of infection. In our Western blots for TAg, we routinely observe two bands using a monoclonal anti-TAg antibody, which are labeled as TAg and TAg* (filled arrowheads). We think these are two forms of TAg because TAg is known to undergo multiple post-translational modifications [23]–[25]. While the total level of ATM remained constant throughout the course of infection, the level of ATM-pS1981 increased dramatically by 2 days post infection (dpi). Concomitant with the increase of ATM-pS1981, there was an induction of NBSI-pS343, γH2AX, Chk2-pT68, RPA32-pS4/8, and p53-pS15, all of which are downstream targets of active ATM-pS1981 [8], [12]. The induction of both ATM-pS1981 and γH2AX was similar to that seen when RPTE cells were treated with IR (data not shown), suggesting that our detection of DDR markers is specific. There was an increase in total p53 levels following BKPyV infection, consistent with previous reports of TAg stabilizing p53 [26]–[28]. In contrast to SV40 [17], BKPyV did not cause degradation of MRN components. In addition, we detected that BKPyV infection resulted in unique γH2AX and Mre11 staining patterns (Figure S1). There was a marked increase in both bright foci and pan-nuclear staining of γH2AX, as well as bright nuclear foci of Mre11. Some, but not all of these foci co-localized with small TAg foci. Together, these results demonstrated that the ATM signaling pathway is activated upon BKPyV infection in RPTE cells.

We next determined whether BKPyV infection led to cell cycle dysregulation (Figure 1B), which is commonly seen with DDR activation. Flow cytometry analysis of cells stained with propidium iodide (PI) showed that starting from 2 dpi, in cells that were infected with BKPyV, there was a gradual increase of a cell population with >G2 DNA content (Figure 1B and 1C). The accumulation of these polyploid cells persisted until 6 dpi (data not shown), whereas in mock-infected cells, most cells were in the G0/G1 phase throughout the time course. These data indicated that multiple rounds of DNA replication can occur within a single cell cycle in BKPyV-infected cells, and are consistent with the activation of the DDR.

### ATM contributes to BKPyV replication

**Figure 2:**
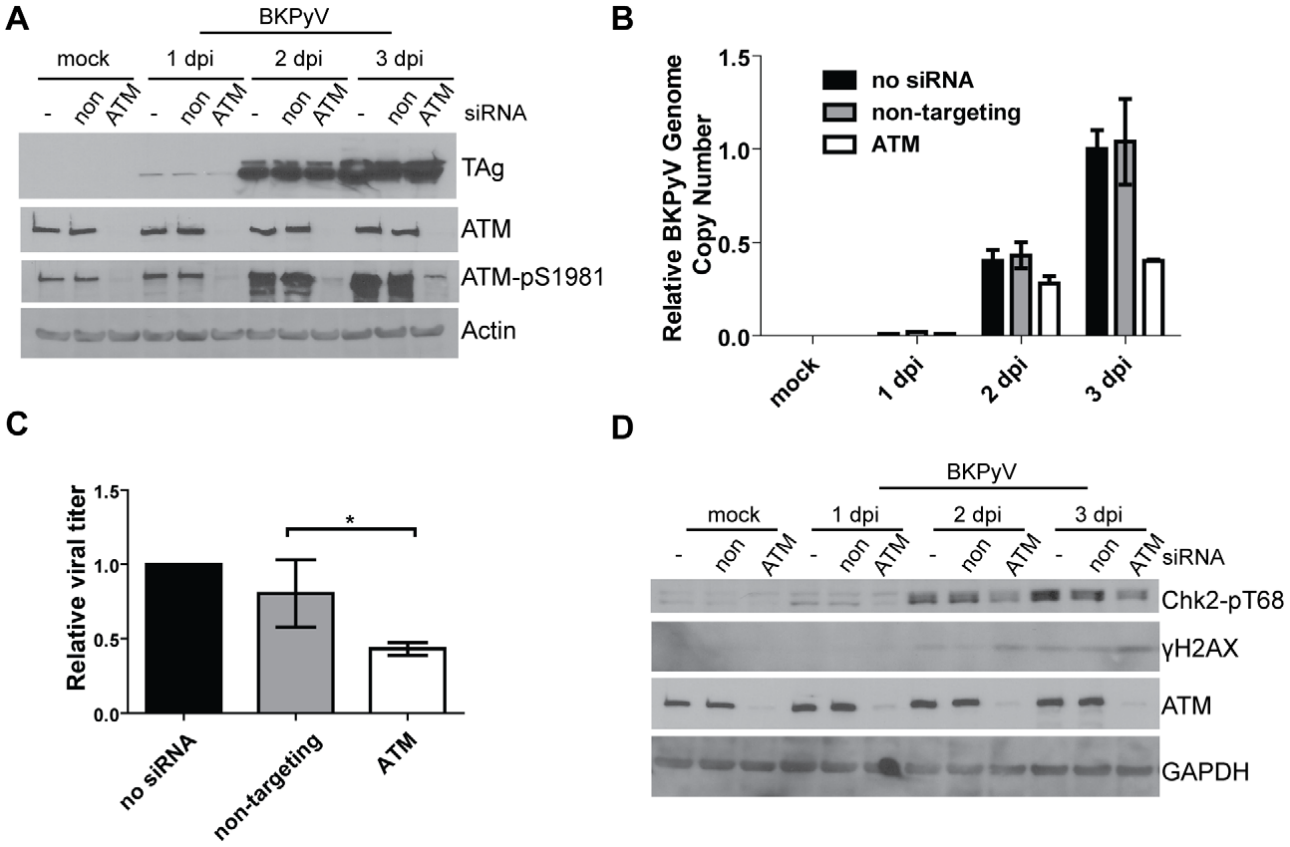
ATM is not required for TAg expression but is required for optimal BKPyV replication. RPTE cells were transfected with no siRNA or the indicated siRNAs followed by infection with BKPyV at an MOI of 0.5 IU/cell at 3 dpt. Non, non-targeting siRNA. (A) Total proteins were harvested at the indicated times post infection and probed for TAg, ATM, ATM-pS1981, and actin by Western blotting. (B) Low molecular weight DNA was extracted from the cells and BKPyV viral DNA was quantified using real-time PCR. All data were normalized to mitochondrial DNA present in the same sample (mitochondrial DNA level was not affected by BKPyV infection; data not shown), and “no siRNA 3 dpi” was arbitrarily set to 1. One representative experiment from two independent experiments is shown; triplicate samples were analyzed in the same assay. Error bars are the SD values. (C) Viral lysates were harvested from the same cells at 2 dpi, and quantified using an IU assay. “no siRNA” was arbitrarily set to 1. Each bar represents the average from three independent experiments and the error bars are the SD values. *, p<0.05. (D) Total proteins were harvested as in (A) and probed for the indicated DDR proteins by Western blotting.

To address the functional roles of the ATM-mediated DDR in productive BKPyV infection, we knocked down ATM using siRNA and assessed the effects of the knockdown on BKPyV infection. RPTE cells were first transfected with siRNAs that targeted ATM, followed by infection with BKPyV when the knockdown of ATM was achieved at 3 days post transfection (dpt) (Figure 2). By Western blotting, ∼90% of the total ATM was knocked down, which was also confirmed by immunoblotting against ATM-pS1981 (Figure 2A and data not shown). The knockdown lasted throughout the course of infection and was not affected by BKPyV replication (Figure 2A). Moreover, knockdown of ATM did not affect the morphology or viability of RPTE cells compared to cells that did not receive siRNA or cells that were transfected with non-targeting siRNA control (data not shown). We first compared the level of TAg in ATM knockdown cells to control cells using immunoblotting. The expression of TAg was similar among all the cells over a 3-day time course, suggesting that ATM was not required for viral early gene expression (Figure 2A). We then examined whether ATM knockdown resulted in a defect in viral DNA replication or infectious progeny production. Low molecular weight DNA was extracted from the samples and real-time PCR was employed to measure the viral DNA load (Figure 2B). The knockdown led to an ∼60% decrease of viral DNA compared to control cells by 3 dpi (Figure 2B). Consistently, we also observed an approximately 50% decrease of viral infectious progeny in ATM knockdown cells (Figure 2C). These data suggested that ATM contributes to optimal BKPyV DNA replication and viral growth.

To ask whether ATM was responsible for the activation of the DDR we observed during BKPyV infection, total proteins from mock- and BKPyV-infected ATM knockdown cells were immunoblotted for Chk2-pT68 and γH2AX and compared to control cells (Figure 2D). ATM knockdown partially abolished the induction of Chk2-pT68 caused by BKPyV infection (Figure 2D), consistent with ATM being the main kinase that phosphorylates Chk2 during the DDR [8], . γH2AX, however, was still induced in BKPyV-infected, ATM knockdown cells (Figure 2D). These data suggested that ATM may not be the sole contributor to the BKPyV-induced DDR.

### ATR contributes to partial DDR activation during BKPyV infection

**Figure 3:**
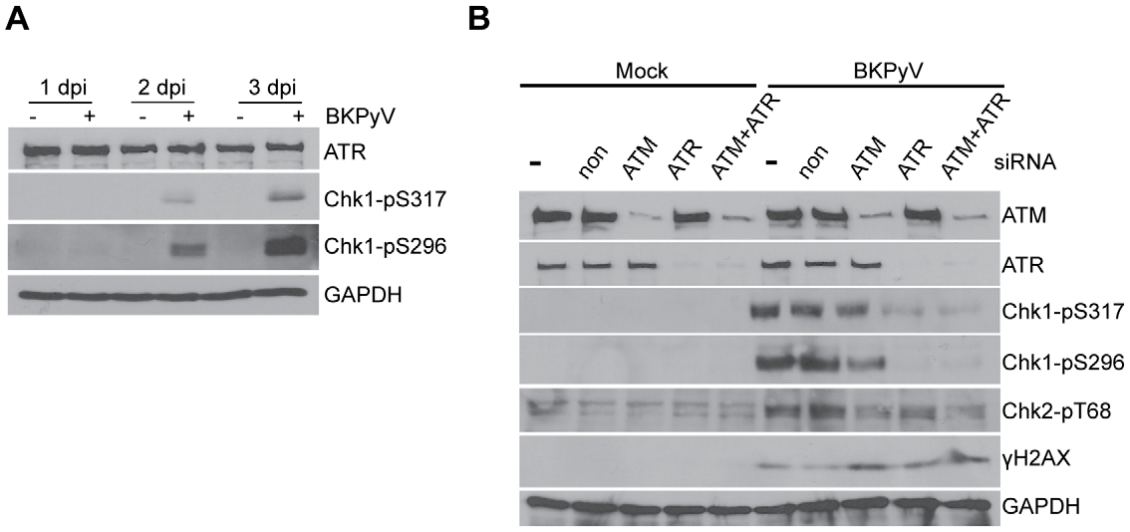
ATR-mediated DDR is activated during BKPyV infection. (A) Total proteins were harvested as described in Figure 1 and probed for the indicated proteins by Western blotting. (B) Cells were transfected with indicated siRNAs and infected as described in Figure 2. Total proteins were harvested at 3 dpi and probed for the indicated proteins.

The fact that ATM knockdown did not completely eliminate the BKPyV-activated DDR suggested that there are other DDR factors involved. Both ATM and ATR share a number of substrates and therefore it is possible that the DDR activation we observed during BKPyV infection was, in part, a result of ATR activation. To determine whether the ATR-mediated DDR is also activated during BKPyV infection, we immunoblotted proteins from mock- and BKPyV-infected RPTE cells for ATR, Chk1-pS317, and Chk1-pS296 (Figure 3A). ATR levels remained relatively unchanged during infection; however, both Chk1-pS317 and Chk1-pS296 increased starting at 2 dpi. Chk1-pS317 phosphorylation is a direct result of ATR activation upon DNA damage, which is followed by Chk1 autophosphorylation on S296 [29]. The strong induction of both Chk1-pS317 and Chk1-pS296 clearly suggested that an ATR-mediated DDR is also activated by BKPyV infection.

Next, we used siRNAs to either singly knock down ATR or doubly knock down both ATM and ATR, and examined the effects of these knockdowns on the DDR activation during BKPyV infection. Quantitative Western blotting showed that the knockdowns were ∼90% complete, and no significant cellular viability changes were noticed in knockdown cells (Figure 3B and data not shown). Having established that, we went on to determine whether ATR single knockdown or ATM+ATR double knockdown could eliminate the BKPyV-induced DDR in RPTE cells. Total proteins from mock- and BKPyV-infected control or knockdown cells were immunoblotted for Chk1-pS317, Chk1-pS296, Chk2-pT68, and γH2AX (Figure 3B). ATM knockdown reduced Chk2-pT68 in infected cells, whereas ATR single or ATM+ATR double knockdown almost completely abolished the induction of both Chk1-pS317 and Chk1-pS296. ATR knockdown, however, did not significantly alter the induction of Chk2-pT68. These data suggested that ATM was mainly responsible for the activation of pChk2 upon BKPyV infection, while ATR was more important in inducing pChk1. γH2AX induction was still evident even in ATM+ATR double knockdown cells (Figure 3B), suggesting that ATM and ATR may not be the only kinases contributing to the phosphorylation of this DDR marker.

### ATR functions in parallel to ATM during BKPyV infection

**Figure 4:**
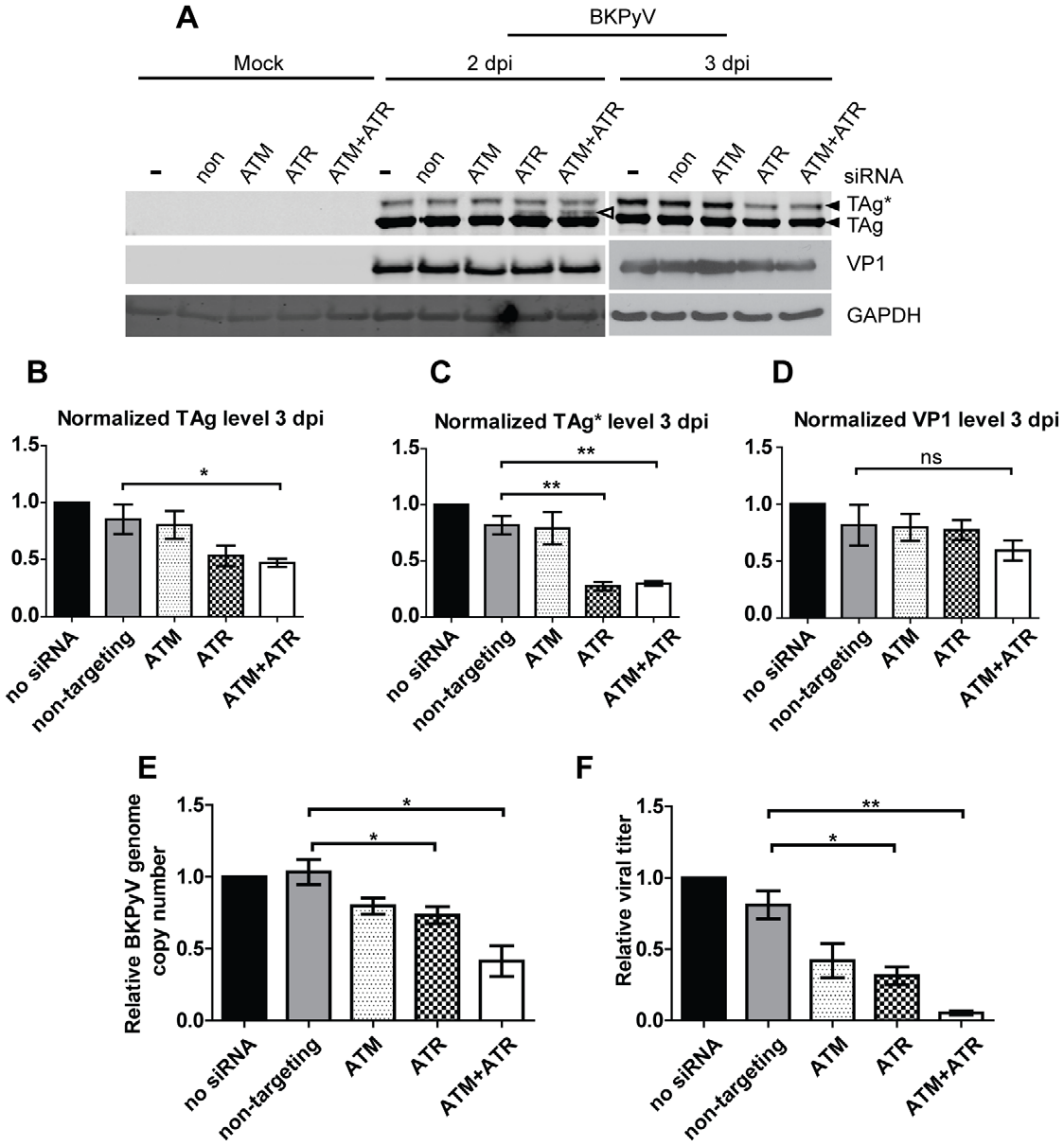
The effect of ATR and ATM knockdown on BKPyV infection. (A) Cells were transfected with indicated siRNAs and infected with BKPyV as described in Figure 2. Total proteins were harvested at the indicated times post infection and probed for the indicated proteins by Western blotting. The open arrowhead points to a third TAg band that appeared in ATR single knockdown and ATM+ATR double knockdown cells at 2 dpi. (B–D) Quantification of the protein bands using the Odyssey Infrared Imaging System. All data were normalized to actin present in the same sample. “no siRNA” was arbitrarily set to 1 in each set. ns, not statistically significant. (E) BKPyV viral DNA and (F) viral titer were quantified as described in Figure 2B and 2C. “no siRNA” was arbitrarily set to 1 in each set. Each bar represents the average from three independent experiments and the error bars are the SD values. *, p<0.05; **, p<0.01.

We then investigated the role that ATR plays alone and in conjunction with ATM during productive BKPyV infection. We first measured viral gene expression in these knockdown cells. At 2 dpi, we did not detect a significant difference in the level of TAg between any knockdown cells and control cells (Figure 4A and S2), but a third TAg band appeared in the ATR and double knockdown cells (Figure 4A, open arrowhead). This band was no longer present at 3 dpi. In contrast to the ATM knockdown alone, at 3 dpi the ATR knockdown displayed a more marked difference in TAg and TAg*. There was about a 50% decrease of TAg in ATR knockdown cells, and an ∼80% decrease of TAg*. The ATM and ATR double knockdown sample had a pattern similar to the ATR knockdown alone (Figure 4B and 4C). None of the knockdowns, however, had a major effect on late gene VP1 expression (Figure 4D and S2). To ensure that we did not saturate the cells with large amounts of BKPyV, we repeated these experiments with a low multiplicity of infection (MOI) (0.01 IU/cell vs. 0.5 IU/cell). Quantitative Western blotting showed that there was no significant difference in either TAg or VP1 levels among all the knockdown and control cells (Figure S3A and S3B) at 2 dpi, suggesting that the lack of difference in TAg and VP1 levels at 2 dpi is not due to compensation from a high MOI infection.

TAg is required for the initiation of viral DNA synthesis and it provides a conducive host replication environment by inactivating the retinoblastoma susceptibility family proteins and p53 [4]. We therefore determined whether a change in TAg in ATR knockdown cells was accompanied by a defect in viral DNA replication. Using quantitative PCR, we found that ATR or ATM knockdown caused a similar decrease in viral DNA synthesis at 2 dpi (Figure 4E). The double knockdown cells, however, exhibited a more severe defect in viral DNA levels at both high and low MOIs (Figure 4E and Figure S3C), suggesting that ATM and ATR function additively to ensure full viral DNA replication. Finally, we measured infectious viral progeny production under these knockdown conditions (Figure 4F). ATR single knockdown led to a slightly larger decrease in viral titer compared to ATM single knockdown. The double knockdown cells displayed a dramatic decrease (∼90%) compared to the control cells.

**Figure 5:**
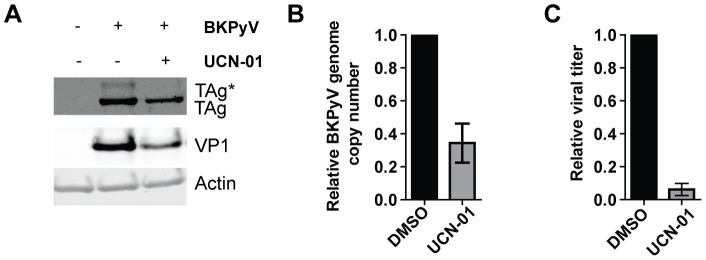
The Chk1 inhibitor UCN-01 blocks BKPyV infection. RPTE cells were infected with BKPyV at an MOI of 0.5 IU/cell. At 1 dpi, UCN-01 was added to the cells and total proteins (A), viral DNA (B), and viral lysate (C) were harvested at 2 dpi and analyzed as in Figure 2. Each bar represents the average from three independent experiments and the error bars are the SD values.

To confirm the importance of the ATR/Chk1 pathway in BKPyV infection, we treated RPTE cells with the Chk1 inhibitor UCN-01 at 1 dpi (at which time the viral genome is delivered into the nucleus) [30]. The presence of UCN-01 slightly reduced TAg and VP1 levels (Figure 5A). Treatment with UCN-01 also resulted in a partial decrease in viral DNA replication and a severe reduction in viral titer (Figure 5B and 5C), consistent with ATR/Chk1 pathway being required for BKPyV productive infection. We have also tested the effect of the ATM inhibitor KU55933; however, this drug seemed to have some off-target effects in RPTE cells and therefore we could not draw conclusions from these experiments (data not shown).

### γH2AX persists in ATM/ATR/DNA-PKcs triple knockdown cells

**Figure 6:**
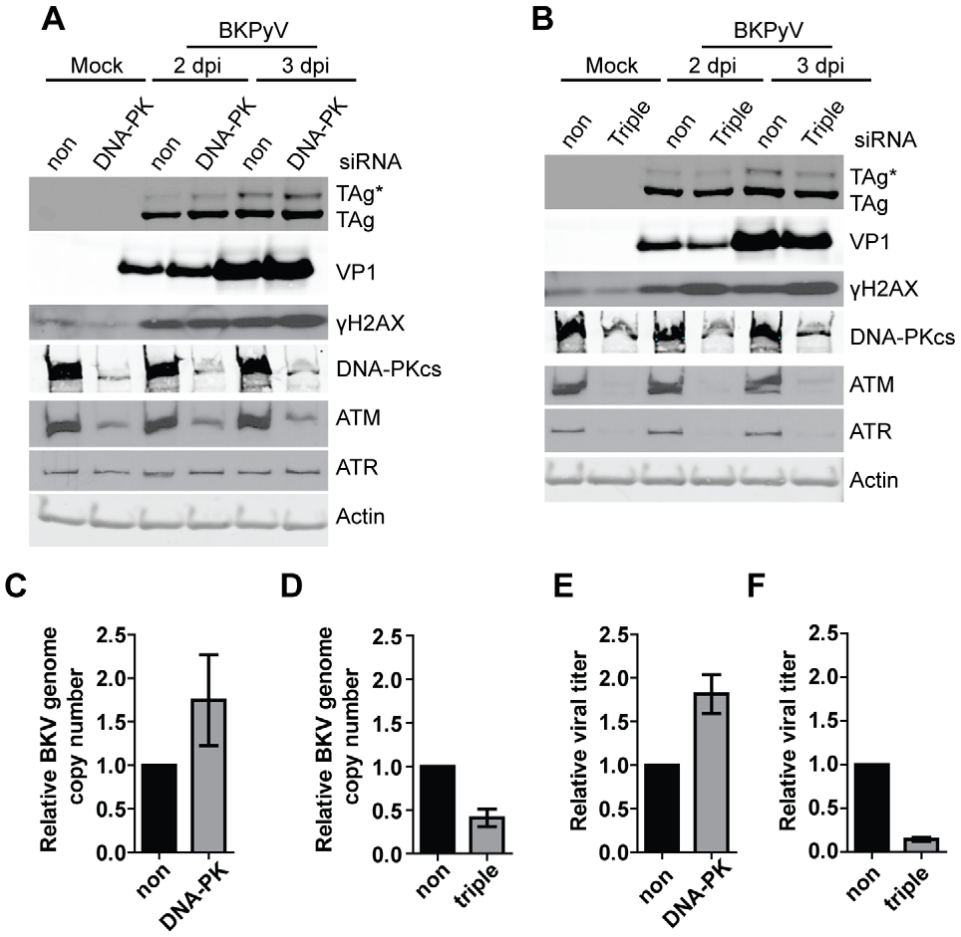
γH2X is activated in ATM, ATR, DNA-PKcs triple knockdown cells. Cells were transfected with indicated siRNAs. (A) DNA-PKcs single knockdown and (B) ATM, ATR, DNA-PK triple knockdown. Total proteins (A, B), viral DNA (C, D), and viral lysate (E, F) were harvested at the indicated times post infection and analyzed as in Figure 2. Each bar represents the average from three independent experiments and the error bars are the SD values.

It has previously been shown that another PI3KK that is involved in nonhomologous end-joining DNA repair, DNA-dependent protein kinase (DNA-PKcs), is able to phosphorylate H2AX independent of ATM and ATR [31], [32]. Additionally, for certain viruses such as adeno-associated virus (AAV), the induction of the DDR in infected cells is mostly mediated through DNA-PKcs [33]. We therefore tested the effect of DNA-PKcs single knockdown and ATM/ATR/DNA-PKcs triple knockdown on BKPyV infection and the induction of γH2AX (Figure 6). The DNA-PKcs-targeting siRNAs efficiently knocked down DNA-PKcs, but they also reduced ATM (Figure 6A). The single knockdown slightly increased TAg, TAg*, and VP1, with a concomitant ∼2 fold increase in viral DNA and viral titer (Figure 6C and 6E). The impact of the ATM/ATR/DNA-PKcs knockdown is similar to that of the ATM/ATR double knockdown. There is an ∼60% drop in viral DNA and a more striking difference in viral titer (Figure 6D and 6F). Surprisingly, with all three kinases absent, we could still detect a strong induction of γH2AX in BKPyV-infected cells (Figure 6B). These data suggested that additional cellular kinases may be involved in the activation of DDR during BKPyV infection.

### ATM and/or ATR knockdown causes severe DNA damage with BKPyV infection

**Figure 7:**
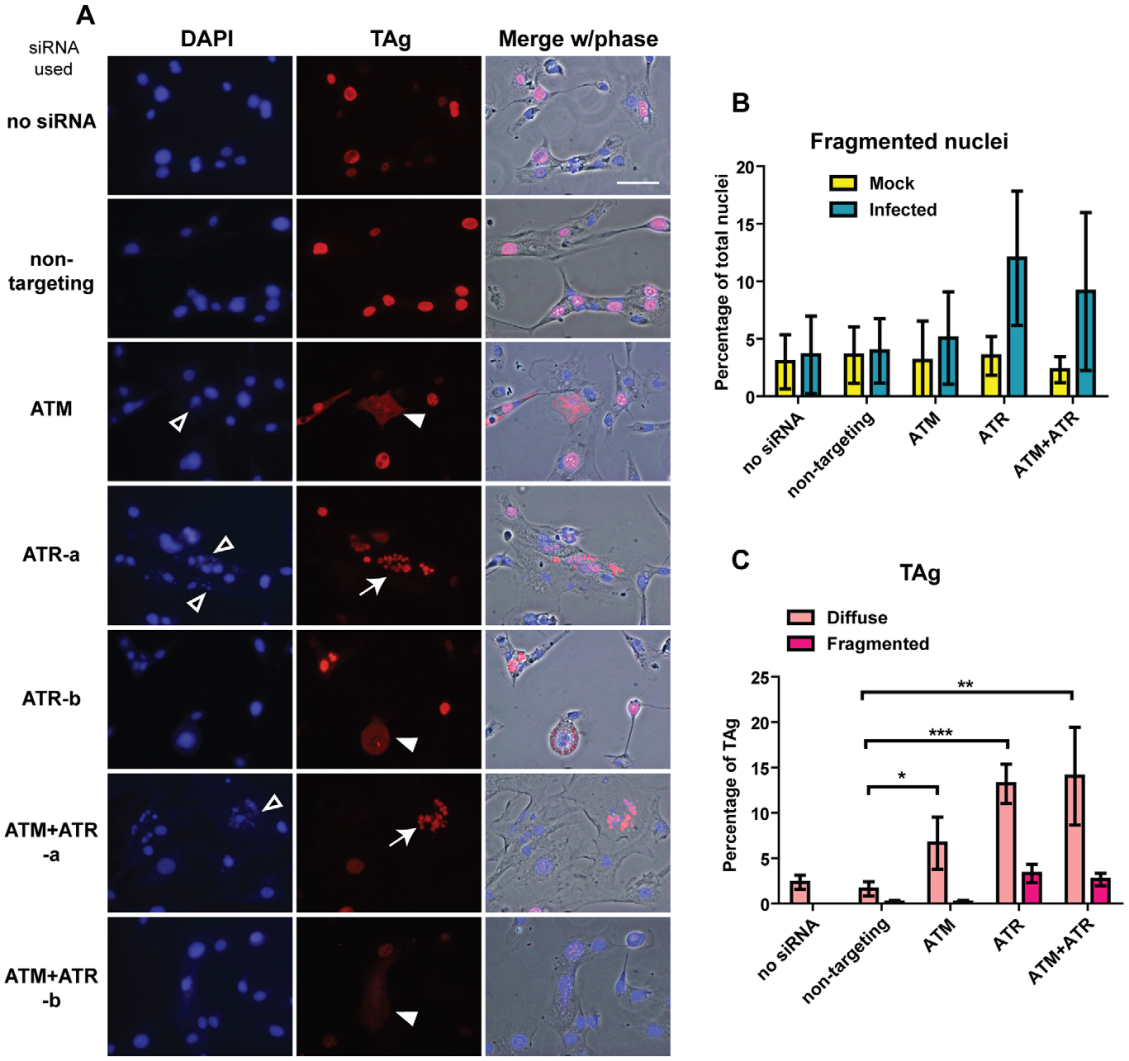
ATM and ATR knockdown leads to aberrant DAPI and TAg staining patterns during BKPyV infection. (A) Cells were transfected with indicated siRNAs and infected with BKPyV as described in Figure 2. Cells were fixed at 3 dpi and stained for DAPI (blue) and TAg (red). Shown are representative epifluorescence pictures and merges with phase contrast pictures. Open arrowheads, fragmented DAPI staining. Filled arrowheads, diffuse TAg staining. Arrows, fragmented TAg. “ATR-a” and “ATR-b” are two different fields of view to show fragmented and diffuse TAg staining, respectively. Similarly, “ATM+ATR-a” and “ATM+ATR-b” are two fields. Scale bar, 50 µm. Bar graphs show the quantitation of fragmented nuclei (B) and aberrant TAg staining (C). Each bar represents the average from three independent experiments (at least 500 nuclei and 100 TAg-positive cells were scored in each sample per independent experiment), and the error bars are the SD values. *, p<0.05; **, p<0.01; ***, p<0.001.

To further dissect the roles that ATM and ATR play during BKPyV infection, we next examined the localization of TAg in knockdown and control cells (Figure 7). In all the mock-infected cells, the nuclei appeared normal judged by DAPI staining (data not shown). With BKPyV infection, however, we observed some abnormal DAPI staining in ATM/ATR single or double knockdown cells (Figure 7A). In these cells, we observed that the nuclei sometimes appeared smaller or fragmented (open arrowheads, Figure 7A, and Figure 7B), which are very similar to micronuclei that arise when chromosomes are broken or damaged [34]. At the same time, we also observed aberrant TAg staining patterns in the knockdown cells (Figure 7A). While TAg appeared mostly nuclear in control cells, in ATM and ATR knockdown cells we often observed cells that had diffuse TAg staining, sometimes throughout the whole cytoplasm (Figure 7A, filled arrowheads, and Figure 7C). In addition, in cells that received ATR siRNA, some of the cells showed fragmented TAg staining patterns (Figure 7A, arrows, and Figure 7C), similar to the fragmented DAPI staining.

**Figure 8:**
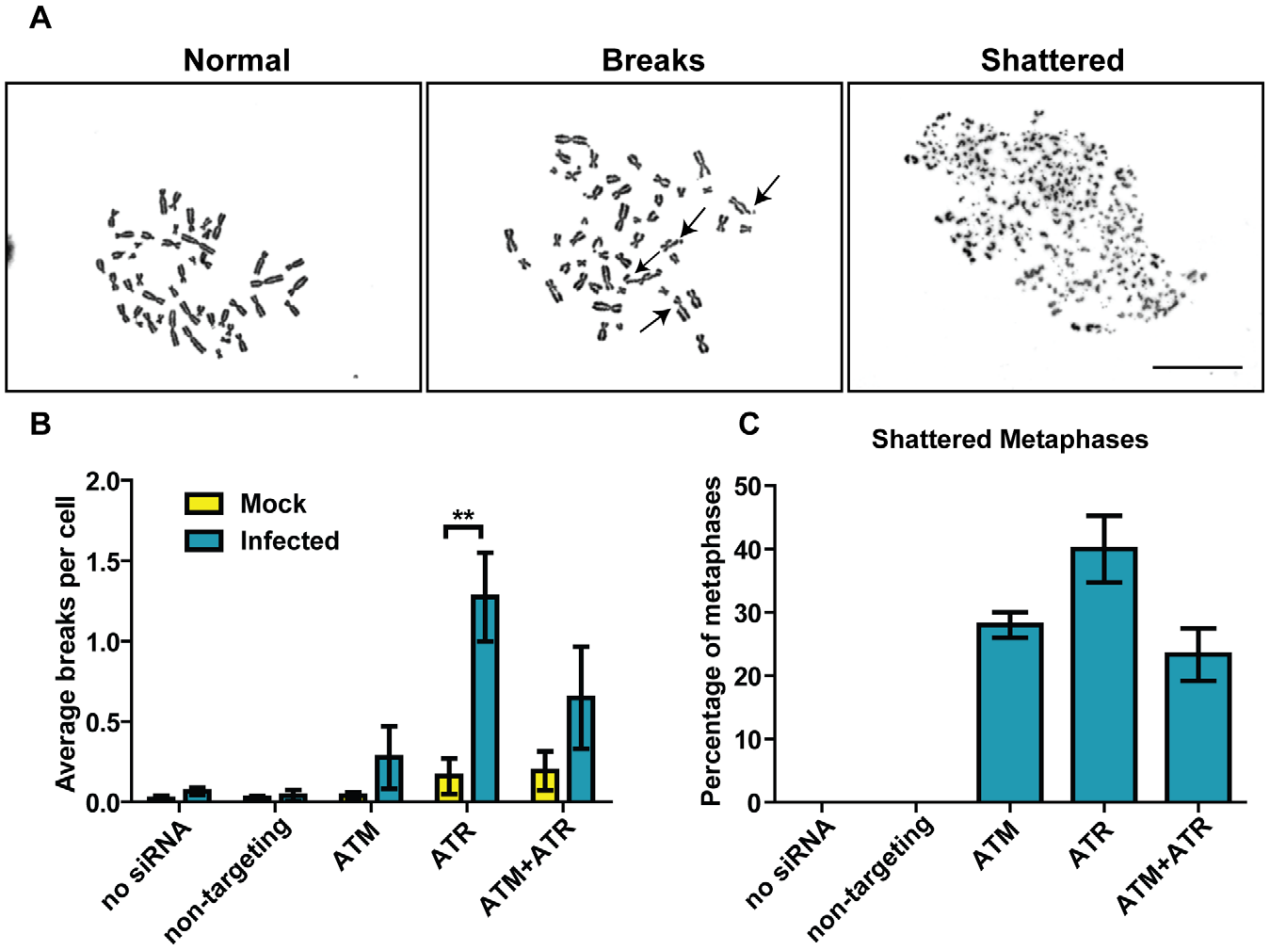
ATM and ATR knockdown results in severe chromosome damage in BKPyV-infected cells. Cells were transfected with indicated siRNAs and infected with BKPyV as described in Figure 2. At 3 dpi, cells were fixed and metaphase chromosomes were prepared as described in the Materials and Methods. (A) Representative pictures of normal chromosomes, chromosomes that contain breaks (arrows point to chromosome breaks), and shattered chromosomes. Scale bar, 20 µm. At least 50 metaphase chromosomes were scored from each sample per independent experiment. Bar graphs show the quantitation of the average number of breaks per cell (among all the non-shattered metaphases) (B) and the percentage of shattered metaphases of all the infected samples (C). Each bar represents the average from three independent experiments and the error bars are the SD values. **, p<0.01.

To confirm that the observed aberrant DAPI and TAg staining is indeed associated with DNA damage, we performed metaphase chromosome analysis in control and knockdown cells, with or without BKPyV infections (Figure 8). In all the mock-infected cells (including all the knockdown cells), or BKPyV-infected cells with no knockdowns, most chromosome spreads were normal. In ATM or ATR knockdown cells, there were a very high percentage of metaphases showing a shattered phenotype, with ATR single knockdown showing the most severe defect (Figure 8A and 8C). These numbers might even be underestimated considering that we could not distinguish between uninfected and infected cells in the metaphase spreads. Shattered chromosomes were never observed in uninfected cells. Among the metaphases that were not shattered, the average number of chromosome breaks per cell was also higher in BKPyV-infected, ATM/ATR knockdown cells (Figure 8B). These data suggested that ATM, and to a greater extent, ATR, contribute to repairing DNA damage that is triggered by BKPyV infection.

## Discussion

**Figure 9:**
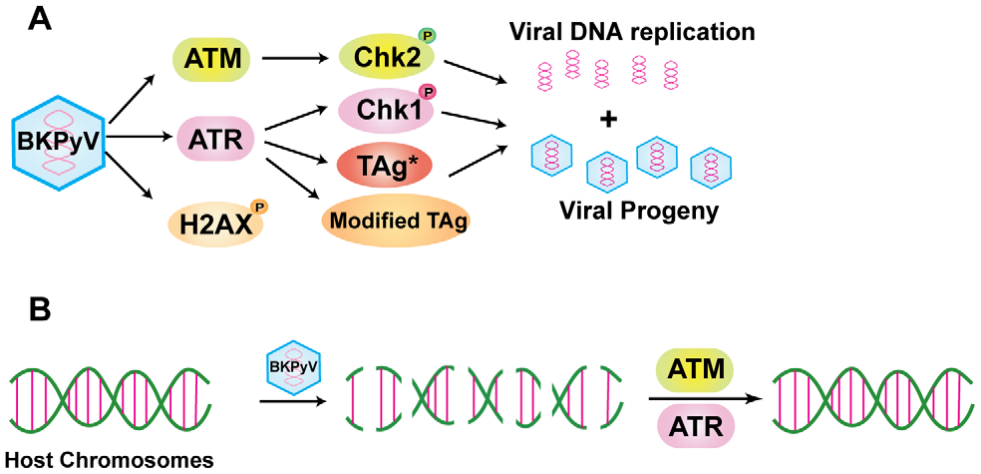
Model of the roles of the DDR during BKPyV infection. (A) Upon BKPyV infection, both the ATM- and ATR-mediated DDR are activated. ATM mainly contributes to the activation of pChk2, whereas ATR mainly activates pChk1. Both ATM and ATR contribute to BKPyV DNA replication and infectious progeny production. ATR may also affect the modification status of TAg. Although γH2AX is induced by BKPyV, the molecular trigger for this induction is unknown. (B) BKPyV infection induces extensive chromosome damage, which is efficiently repaired with the aid of both ATM and ATR.

In this report we characterized the relationship between the ATM- and ATR-mediated DDR and lytic BKPyV infection. Our results indicated that both branches of the DDR were activated by BKPyV; moreover, ATM and ATR functioned in parallel and contributed to the activation of individual DDR pathways (Figure 9A). In particular, ATM was mainly responsible for activating pChk2, whereas ATR was more important in activating pChk1 and also might be important for regulating TAg and its modified forms. Our results showed that both ATM and ATR were required to achieve maximal viral DNA replication and infectious progeny production. This is the first such report for any polyomavirus family member to our knowledge. Knocking down each individual kinase resulted in a partial defect in viral replication and the double knockdown had a more dramatic effect. When comparing ATM and ATR double knockdown cells to control cells, the degree of decrease in viral titer was consistently greater than the decrease in viral DNA, suggesting that there may be some post-DNA replication regulation that involves both ATM and ATR function. For example, ATR may be involved in alleviating the DNA replication stress generated during the resolution of the two daughter circular BKPyV chromosomes, and therefore ATR knockdown caused a more marked defect in viral progeny production than in DNA replication. Our results also showed that in the absence of ATM or ATR, severe chromosome damage accumulated upon BKPyV infection. These data point out that another possible function of ATM and ATR during BKPyV infection is to help repair the DNA damage caused by BKPyV (Figure 9B).

The exact role of ATM during polyomavirus early gene expression remains unclear, but it may be different depending on the specific virus and cell type. For example, one report showed that ATM knockdown in African green monkey CV1 cells reduced the level of SV40 TAg-pS120, but it did not have much effect on the total level of TAg [16]. In another report using BSC40 monkey kidney cells, the ATM inhibitor KU55933 reduced total SV40 TAg levels [17]. Our ATM knockdown data suggest that ATM is not required for TAg expression during BKPyV infection. Consistent with this, BKPyV TAg expression was similar in wild-type and ATM-knockout mouse embryonic fibroblast cell lines (data not shown). Unfortunately we cannot study the entire BKPyV life cycle in mouse cells due to a block to DNA replication [35].

Our data demonstrate the importance of ATM and ATR during BKPyV DNA replication, DDR activation, and progeny production. It is possible that ATM, ATR and downstream effectors directly participate in replication-related events. For example, ATM has been reported to phosphorylate SV40 TAg *in vivo* and therefore is required for optimal SV40 replication [16]. In our ATR single knockdown or ATM+ATR double knockdown cells, we observed a decrease in a modified form of TAg (TAg*) at 3 dpi, and the appearance of a third band of TAg at 2 dpi (Figure 4A). Preliminary experiments indicated that phosphatase treatment did not affect the level of TAg or TAg* (data not shown), but this does not definitively rule out the possibility that TAg* may represent a phosphorylated form of TAg. Additionally, proteins involved in homologous recombination or the Fanconi anemia pathway (required for repair of stalled replication forks) have been found to be necessary for SV40 replication [18]. It is possible that DDR proteins that are downstream of ATM and ATR directly contribute to BKPyV DNA replication. Alternatively, instead of being directly involved in viral replication, ATM and ATR may participate indirectly in BKPyV infection by affecting cell cycle status. For example, ATM and ATR activation leads to G2 arrest in JCPyV-infected human neuroblastoma cells and oligodendrocytes [19]. It is hypothesized that G2 arrest contributes to JCPyV replication by maintaining the cellular replication machinery and preventing mitosis [19]. Our cell cycle analyses also showed that BKPyV infection of RPTE cells results in the accumulation of polyploid cells, consistent with the idea that mitosis is inhibited to allow for maximal DNA replication.

A number of DDR proteins including γH2AX and Mre11 were re-localized into small TAg-containing foci during BKPyV infection. Similar co-localization between DDR proteins and TAg foci is seen during SV40 infection, although the MRN proteins are eventually degraded during SV40 infection [17], [18]. It has been hypothesized that these foci represent sites of viral DNA replication because they also contain proteins that are required for viral replication including RPA and Pol-prim, but not Pol-prim-associated host replication factor [17]. In addition to the small, bright foci of γH2AX, there was also an increase in pan-nuclear staining of γH2AX in BKPyV-infected cells. Although the functional significance of this increase is not clear, it has previously also been reported in other DNA virus infections such as adenovirus and AAV [33], [36], [37]. It is thought that this pan-nuclear staining increase may represent modification of histones on cellular chromatin and that the modification can be stimulated by viral replication.

Intriguingly, the induction of γH2AX by BKPyV still occurred in ATM, ATR, and DNA-PKcs triple knockdown cells, suggesting that there might be other cellular kinase(s) responsible for phosphorylating this molecule. Alternatively, it is possible that BKPyV infection alters a cellular phosphatase activity that, together with residual PI3KK activity due to incomplete knockdown, leads to an increase in the steady state level of γH2AX. One phosphatase candidate is PP2A, which has been demonstrated to dephosphorylate γH2AX in an ATM-, ATR-, and DNA-PKcs-independent manner [38]. Polyomavirus small T antigen is well known for its interaction with PP2A and its ability to inhibit PP2A enzymatic activity [39]. It will be interesting to determine the mechanism of γH2AX induction and its functional significance during BKPyV infection in the future.

What triggers the activation of both the ATM- and ATR-mediated DDR during BKPyV infection requires more careful examination. It is possible that a viral protein(s) alone is able to achieve the induction. For example, expression of SV40 TAg without a viral replication origin in normal human BJ/tert fibroblasts induces both an ATM- and ATR-mediated DDR, and this induction is dependent on the interaction of TAg with Bub1, a mitotic spindle checkpoint kinase [40]. Moreover, polyomavirus TAg alone is able to induce cellular DNA damage as judged by comet assays and cytogenetic analyses [18], [41]–[44]. For JCPyV, the ability of TAg to associate with cellular DNA is important for TAg induction of G2 cell cycle arrest [19]. It is also possible that either incoming or replicating viral genomes serve as the trigger for the DDR. For example, both wild-type and UV-inactivated AAV2, but not recombinant AAV2 vectors, are capable of inducing a DDR, suggesting that it is the viral DNA sequence, but not the viral capsid, that is responsible for the activation of DDR [37].

In conclusion, our results demonstrate the unique activation of various DDR components upon BKPyV infection and the essential roles of both ATM and ATR for viral replication and growth. The study of BKPyV infection and the DDR not only reveals novel knowledge about the cellular pathways with which the virus needs to interact in order to complete the lytic life cycle, but may also have important clinical implications for BKPyV reactivation and its related disease. For example, BKPyV reactivation is a severe problem in bone marrow transplant patients, who might have experienced radiation as part of the preparative regimen. Thus, research focusing on BKPyV and DDR may shed light on the analysis of the causality of BKPyV reactivation in these patients.

## Materials and Methods

### Cell culture, viruses, and infections

RPTE cells (Lonza) were maintained in renal epithelial cell growth medium (REGM) as previously described [45]. All cells were grown at 37°C with 5% CO_2_ in a humidified incubator.

BKPyV (Dunlop) was grown, purified, and titered using an infectious unit (IU) assay as previously described [46]. For infections, RPTE cells were pre-chilled for 15 min at 4°C. The cells were then exposed to purified BKPyV diluted in REGM at the indicated MOIs and incubated for 1 h at 4°C. Infection was initiated by replacing the viral inoculum with pre-warmed REGM and transferring the cells to 37°C. Total cell proteins and viral lysates were harvested as previously described [45].

### Drug treatments

UCN-01 (Sigma) was reconstituted according to the manufacturer's recommendations. The drug was added at 1 dpi at 100 nM and was left on for the time of the experiment. A cell metabolism WST-1 assay (Roche) was used to ensure that the drug treatment did not cause significant cytotoxic effects (data not shown).

### Western blotting and quantification

Total cell proteins were harvested, quantified, and immunoblotted as previously described [46]. For quantitative blots using the Odyssey Infrared Imaging System, the membrane was processed according to the manufacturer's instructions (LI-COR). The membrane was scanned using the Odyssey Infrared Imaging system, and the relevant bands were quantified using the Odyssey software. See Table S1 for a list of antibodies and concentrations used in this study.

### Cell cycle analysis

Mock or BKPyV-infected cells were trypsinized, resuspended in PBS, and fixed with 100% cold ethanol. DNA was labeled with 50 µg/ml PI+100 µg/ml RNAse in PBS at room temperature for 30 min. Samples were analyzed with a BD FACSCalibur flow cytometer and the cell cycle data were modeled using ModFit LT software.

### Immunofluorescence microscopy

At the indicated times post infection, RPTE cells were fixed and immunostained as previously described [47] with antibodies listed in Table S1. For standard fluorescence microscopy, samples were examined using an Olympus BX41 microscope with a Plan 40×/0.65 objective and processed using the Olympus DP manager software. For laser-scanning confocal microscopy, all images were obtained using a Zeiss LSM 510 confocal microscope with a 63×/1.2 objective and 1 µm optical section. Images were analyzed and processed using LSM image browser (Zeiss).

### siRNA knockdown

siRNA ON-TARGET plus SMART pools were purchased from Thermo Scientific Dharmacon: Non-targeting (D-001810-10-05); ATM (L-003201-00-0005); ATR (L-003202-00-0005); and DNA-PK (L-005030-00-0005). siRNAs were resuspended in 1× siRNA buffer (Dharmacon) to 20 µM stocks. RPTE cells were reverse transfected with indicated siRNAs using Lipofectamine RNAiMAX transfection reagent (Invitrogen) according to manufacturer's instructions. siRNAs were diluted in REGM without serum or antibiotics and mixed with Lipofectamine RNAiMAX (2–4 µl per well of a 12 well plate, or 33 µl per T75 flask). The complexes were allowed to form at room temperature for 15 min, followed by the addition of RPTE cells (60,000 cells per well, or 660,000 cells per flask). The optimal final concentrations of siRNA were determined empirically. ATM and DNA-PK siRNAs were used at 10 nM, and ATR siRNA was used at 20 nM. For double or triple knockdowns, non-targeting siRNAs were added to ensure that the total concentrations of siRNAs in all the samples were the same. Transfection complexes were washed out 1 dpt and replaced with REGM containing serum and antibiotics [45]. The cells were infected with BKPyV at 3 dpt as described above. For some batches of RPTE cells, siRNA transfection resulted in uneven cell death among different wells. Under these circumstances, cells were trypsinized, counted, and re-plated prior to infection to ensure that same number of cells were present in all samples for infection.

### Real-time PCR

To quantify the viral DNA load in cells, low molecular weight DNA was isolated using a modified Hirt protocol [47], real-time PCR reactions were performed, and data were analyzed as previously described [47].

### Metaphase chromosome preparations

Cells were harvested for chromosome preparations with a modified protocol [48]. Briefly, cells were treated with colcemid (50 ng/ml) for 1 h followed by an 18 min incubation in 0.8% sodium citrate at 37°C and multiple changes of Carnoy's fixative (3∶1 methanol∶acetic acid). Cells were dropped onto slides and slides were baked overnight at 55°C before staining with Giemsa (Sigma). Metaphase chromosomes were observed using an Olympus BX41 microscope with a Plan 100×/1.25 oil objective or a Nikon OPTIPHOT microscope with a Plan 100×/1.40 oil objective.

## Supporting Information

Figure S1 BKPyV infection results in a re-distribution of DDR markers. RPTE cells were mock infected or infected with BKPyV as in Figure 1. Confocal images of cells that were fixed at the indicated times post infection and immunostained for (A) γH2AX (green) and TAg (red) and (B) Mre11 (green) and TAg (red) are shown. The inserts (magnified 2 fold) in (B) show the co-localization between Mre11 and TAg foci. Scale bar, 10 µm.
(TIF)

Figure S2 ATM and ATR do not affect TAg or VP1 levels during early infection. Cells were transfected with indicated siRNAs and infected with BKPyV as described in Figure 4. Total proteins were harvested at 2 dpi and probed for TAg and VP1. Quantitation of TAg (A) and VP1 (B) was performed as described in Figure 4. No samples showed statistically significant differences compared to non-targeting controls.
(TIF)

Figure S3 The effect of ATM and ATR knockdown on BKPyV infection during low MOI infection. Cells were transfected with indicated siRNAs and infected with BKPyV at an MOI of 0.01 IU/cell. TAg (A) and VP1 (B) levels were quantified as described in Figure 4. No samples showed statistically significant differences compared to non-targeting controls. (C) Relative BKPyV DNA load was quantified as described in Figure 2B. *, p<0.05.
(TIF)

Table S1 List of antibodies used in this study. Abbreviations: IFA, immunofluorescent analyses; WB, Western blotting; HRP, horseradish peroxidase.
(DOCX)
