# Supplementary figures and images for: Roles of ATM and ATR-Mediated DNA Damage Responses during Lytic BK Polyomavirus Infection
Source: PLoS Pathog. 2012 Aug 30;8(8):e1002898. doi: 10.1371/journal.ppat.1002898 (PMC3431332; doi:10.1371/journal.ppat.1002898)

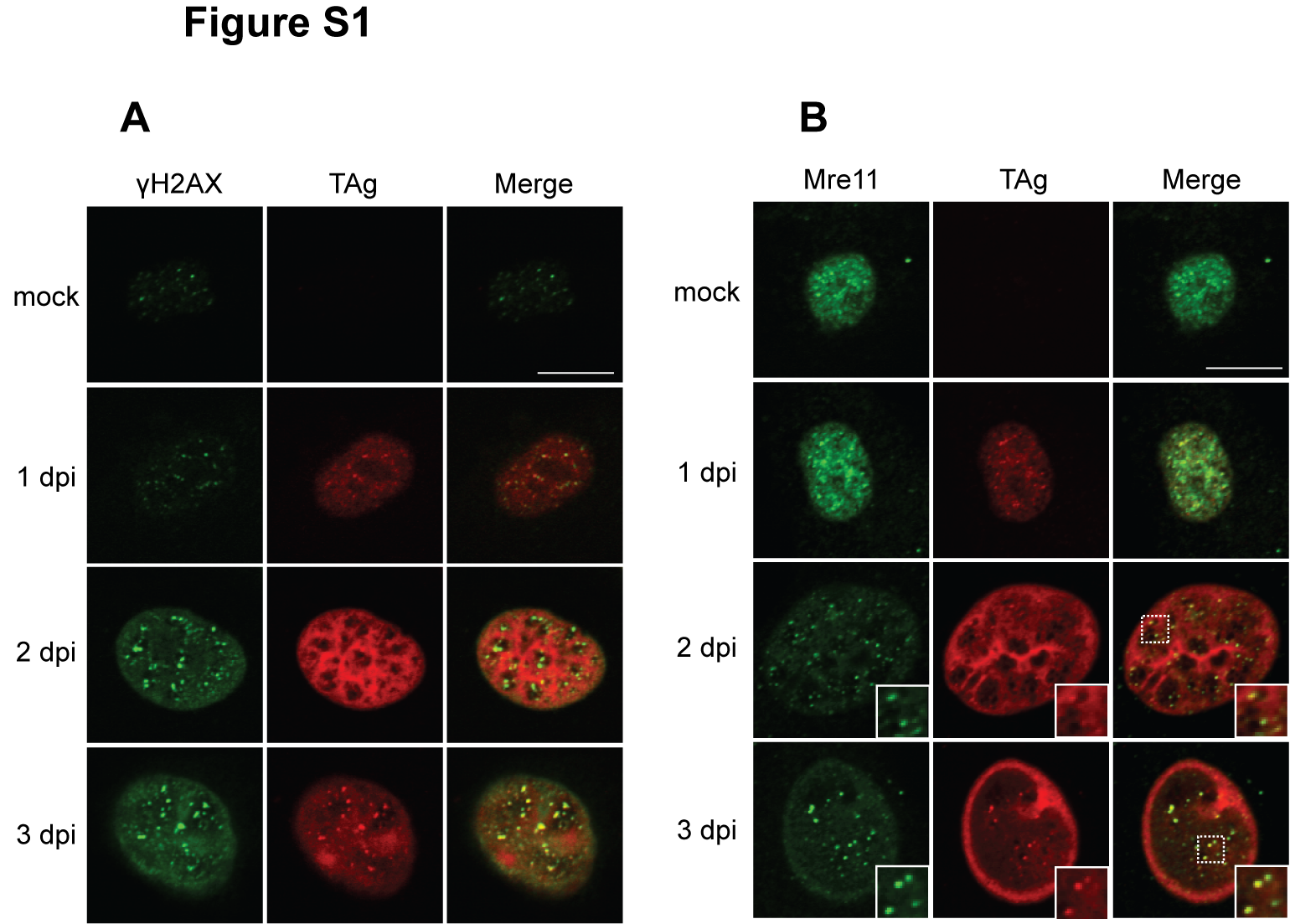

Supplement: Figure S1 — BKPyV infection results in a re-distribution of DDR markers. RPTE cells were mock infected or infected with BKPyV as in Figure 1. Confocal images of cells that were fixed at the indicated times post infection and immunostained for (A) γH2AX (green) and TAg (red) and (B) Mre11 (green) and TAg (red) are shown. The inserts (magnified 2 fold) in (B) show the co-localization between Mre11 and TAg foci. Scale bar, 10 µm. (TIF) [file ppat.1002898.s001.tif]

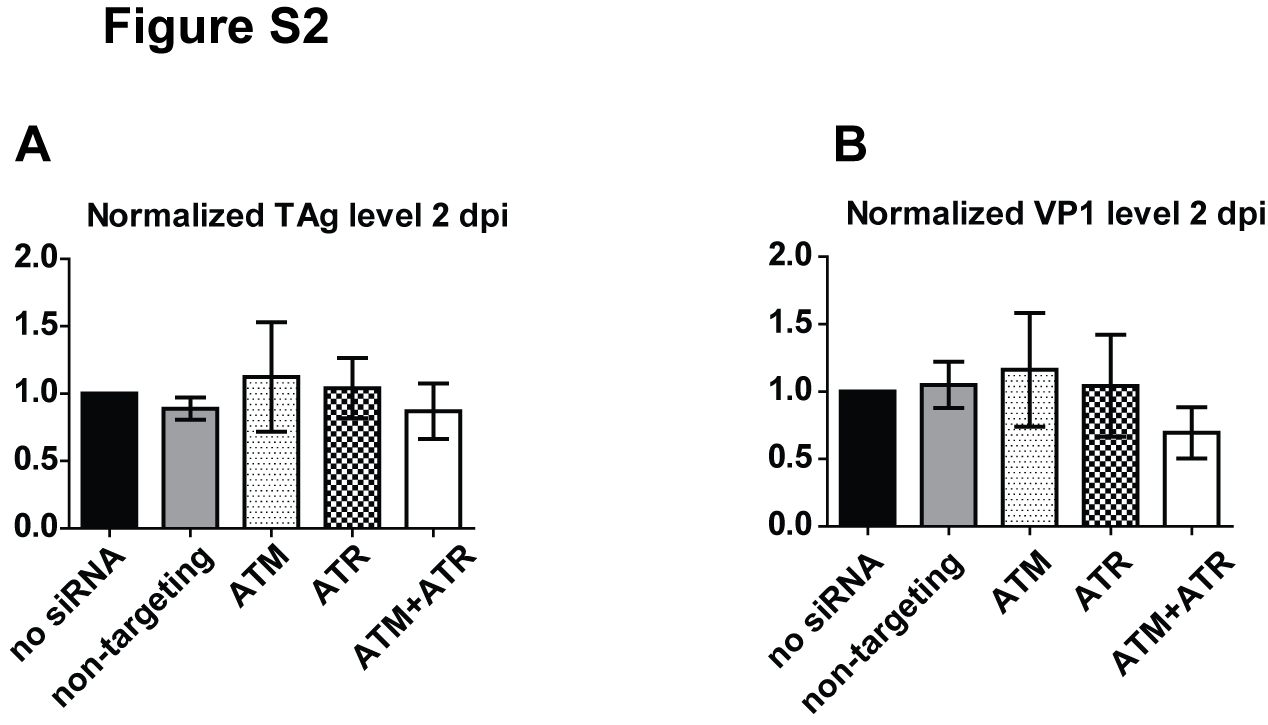

Supplement: Figure S2 — ATM and ATR do not affect TAg or VP1 levels during early infection. Cells were transfected with indicated siRNAs and infected with BKPyV as described in Figure 4. Total proteins were harvested at 2 dpi and probed for TAg and VP1. Quantitation of TAg (A) and VP1 (B) was performed as described in Figure 4. No samples showed statistically significant differences compared to non-targeting controls. (TIF) [file ppat.1002898.s002.tif]

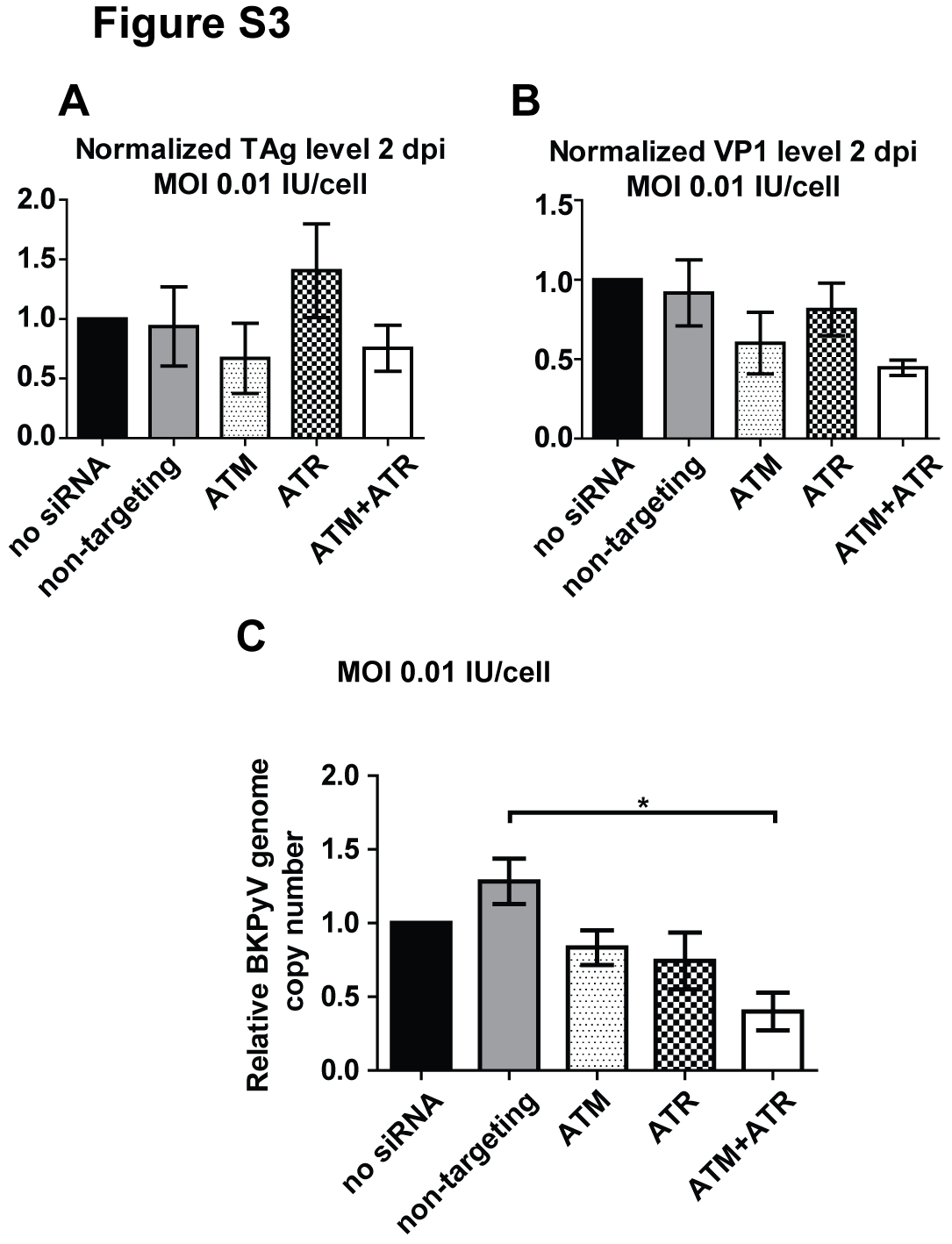

Supplement: Figure S3 — The effect of ATM and ATR knockdown on BKPyV infection during low MOI infection. Cells were transfected with indicated siRNAs and infected with BKPyV at an MOI of 0.01 IU/cell. TAg (A) and VP1 (B) levels were quantified as described in Figure 4. No samples showed statistically significant differences compared to non-targeting controls. (C) Relative BKPyV DNA load was quantified as described in Figure 2B. *, p<0.05. (TIF) [file ppat.1002898.s003.tif]
