# Supplementary material for: Roles of ATM and ATR-Mediated DNA Damage Responses during Lytic BK Polyomavirus Infection
Source: PLoS Pathog. 2012 Aug 30;8(8):e1002898. doi: 10.1371/journal.ppat.1002898 (PMC3431332; doi:10.1371/journal.ppat.1002898)
Supplement: Table S1 — List of antibodies used in this study. Abbreviations: IFA, immunofluorescent analyses; WB, Western blotting; HRP, horseradish peroxidase. (DOCX) [file ppat.1002898.s004.docx]

| **Name** | **Source** | **Dilution** | **Purpose** |
| --- | --- | --- | --- |
| TAg (pAb416) | [[46](#_ENREF_46)] | 1:200 | IFA |
|  |  | 1:3,000 | WB |
| ATM | Cell signaling, 2873 | 1:1,000 | WB |
| ATM-pS1981 | Epitomics, 2152-1 | 1:2,000 | WB |
| NBSI | Novus, NB100-143 | 1:300 | IFA |
|  |  | 1:2,000 | WB |
| NBSI-pS343 | GeneTex, GTX82952 | 1:1,000 | WB |
| γH2AX | Cell signaling, 9718 | 1:200 | IFA |
| γH2AX | Novus, NB100-384 | 1:2,000 | WB |
| Mre11 | Novus, NB100-142 | 1:300 | IFA |
|  |  | 1:5,000 | WB |
| Chk2-pT68 | Cell signaling, 2661 | 1:1,000-2,000 | WB |
| Chk1-pS296 | Cell signaling, 2349 | 1:1,000-2,000 | WB |
| Chk1-pS317 | Cell signaling, 2344 | 1:1,000 | WB |
| Rad50 | Novus, NB100-154 | 1:5,000 | WB |
| RPA32 | GeneTex, GTX70258 | 1:2,500 | WB |
| RPA32-pS4/8 | Bethyl, A300-245A | 1:2,000 | WB |
| p53 | Oncogene, OP43 | 1:2,000 | WB |
| p53-pS15 | Cell signaling, 9284 | 1:2,000 | WB |
| ATR | Novus, NB100-323 | 1:1,000 | WB |
| VP1 | [[46](#_ENREF_46)] | 1:5,000 | WB |
| DNA-PKcs | Thermo Scientific, MS-423 | 1:1,000 | WB |
| GAPDH | Abcam, ab9484 | 1:5,000-10,000 | WB |
| β-Actin | Cell signaling, 4967 | 1:5,000 | WB |
| HRP-conjugated sheep anti-mouse IgG | Amersham | 1:2,000-5,000 | WB |
| HRP-conjugated donkey anti-rabbit IgG | Amersham | 1:2,000-5,000 | WB |
| Alexa Fluor 488 goat anti-rabbit IgG (H+L) | Invitrogen | 1:200 | IFA |
| Alexa Fluor 594 goat anti-mouse IgG (H+L) | Invitrogen | 1:200 | IFA |
| Goat anti-rabbit IRDye 680LT | LI-COR | 1:10,000 | WB |
| Goat anti-mouse IRDye 800CW | LI-COR | 1:10,000 | WB |
